# EXPLORING STRATEGIES FOR IMPROVED COORDINATION AND INNOVATION IN TRANSPORTATION FOR OLDER PEOPLE

**DOI:** 10.1093/geroni/igac059.704

**Published:** 2022-12-20

**Authors:** Nina Silverstein, Taylor Jansen, Alycia Bayne

## Abstract

Communities across the nation have outgrown their capacities to meet the transportation needs of older people. While a struggle in the past, for many communities it now is a crisis given an increase in older people with impairments in critical driving skills that lead to driving cessation and/or limitations that preclude them from successfully navigating public transit. It is time for new strategies and innovations to meet these growing challenges. Jansen will describe a pilot study of a regional transit authority’s use of customized software with four Councils on Aging to increase riders’ trips and destinations, improve efficiencies in reporting and dispatching, and increase shared rides across the communities. Gleason will present results of a qualitative study of paratransit managers across the U.S. and their attempts to innovate and adapt to evolving market expectations spurred on by the emergence of transportation network companies (TNC) like Uber and Lyft. Schwartz describes a mixed-methods study of Project TRIP, a rural transportation program designed to increase access to healthcare and other locations for low income and vulnerable populations in rural eastern North Carolina. Lynott introduces AARPs RideSheet, an open-source ride scheduling software application designed for small, demand-responsive transportation providers that incorporates a transactional data specification (TDS) enabling two or more providers to interoperate more efficiently, improving service for their clients. Bayne concludes as discussant bringing in her own work with the CDC on barriers and facilitators to ride sharing and reflecting on the themes presented. Transportation and Aging and Qualitative Research IGs collaboration.

